# Inorganic arsenic contamination and the health of children living near an inactive mining site: northern Thailand

**DOI:** 10.17179/excli2022-4922

**Published:** 2022-07-26

**Authors:** Sarun Kunwittaya, Nootchanart Ruksee, Thirata Khamnong, Athiwat Jiawiwatkul, Nonthasruang Kleebpung, Vasunun Chumchua, Adisak Plitponkarnpim, Chutikorn Nopparat, Kannika Permpoonputtana

**Affiliations:** 1 National Institute for Child and Family Development, Mahidol University, Nakhon Pathom 73170, Thailand; 2 Faculty of Medicine Ramathibodi Hospital, Mahidol University, Bangkok 10400, Thailand; 3 Innovative Learning Center, Srinakharinwirot University, Sukhumvit 23, Bangkok, 10110, Thailand

**Keywords:** children, inorganic arsenic, mining industry

## Abstract

Arsenic toxicity is a global health problem affecting millions of people. Contamination is caused by arsenic from natural geological sources leaching into aquifers, contaminating drinking water, and may also be caused by mining and other industrial processes. Acute arsenic poisoning is associated with nausea, vomiting, abdominal pain, and severe diarrhea. Chronic arsenic toxicity results in multisystemic diseases leading to central nervous system (CNS) impairments such as cognitive or intellectual deficits in children. Over the past ten years, arsenic contamination has been reported in northern Thailand. The Ministry of Public Health; Thailand, Forensic Science Institute Thammasat University, and the Research Center to Promote Safety and Prevent Injuries in Children at the Ramathibodi Hospital compiled a report on the health impact of the population within a 10 kilometer radius around a mine tailing in the Phichit, Phitsanulok, and Phetchabun Provinces of Thailand. It showed that more than 30 % of children (aged 8-13 years) had higher than normal arsenic contamination levels based on the Agency for Toxic Substances and Disease Registry (ATSDR). After the publication of that report, the mine was temporarily closed in 2016. Based on this data, this research aimed to follow arsenic contamination after the mining operation had stopped operation for three years. The study showed that 4.5 % of school aged children had levels of inorganic arsenic in their urine, higher than the normal range (ATSDR), showing clearly that inorganic arsenic contamination is still above the normal range in children living near an inactive mining site**. **Therefore, monitoring heavy metal contamination in Thailand and the health effects on vulnerable children who live near mines during regular operation or after being temporarily suspended can prevent and mitigate possible health impacts.

## Introduction

Mining and associated industries can cause heavy metal contamination in the environment and communities nearby even after the facilities have closed or work has ceased. Exposure to various heavy metals from the mining industry can occur via inhalation, ingestion of windblown soil dust, and ingestion of contaminated drinking water (Miranda et al., 2016[12]). 

In Thailand, heavy metal contamination, particularly arsenic, had been reported in 1987 at the Ron Pibul district of the Nakorn Srithammarat Province in the southern region of Thailand. It caused more than 1,000 people to be contaminated with arsenic and clinical symptoms including skin keratosis, a melonosis with “raindrop in the dust” appearance, skin cancer, weakness, mild fever, anemia, numbness, and dysfunction of upper and lower extremities were observed. The mining industry finally closed in 1994 (Vitayavirasak et al., 2005[20]). 

It is assumed that exposure to arsenic occurred through drinking contaminated water from mines and using contaminated ground water (Williams et al., 1996[25]). The previous review showed that widespread contamination of ground water by arsenic has been reported in many countries such as Bangladesh, West Bengal, China, Taiwan, Thailand, Ghana, Argentina, Chile, Mexico, Hungary, Canada, the United Kingdom, and areas of the United States (Kapaj et al., 2006[9]). In Thailand, there have been reports of arsenic contamination from surface drainage and leachate from mine waste causing the distribution of arsenic over an area of about 12 square kilometers (BOE, 2010[2]). 

Exposure to heavy metals such as arsenic in children is a public health concern because they are one of the most susceptible groups in the population with bodies that are still developing (Hines et al., 2010[6]). In addition, children are more likely to play outdoors or on the floor and inadvertently can ingest dust that adheres to hands or other objects. For example, in the community near a former copper smelter in Montana, Hwang and colleagues found a significant correlation between arsenic concentrations in residential soils and urinary arsenic in children less than 72 months old, with the highest correlation for soils in bare yards (Hwang et al., 1997[8]). 

Arsenic exposure at an early age may also lead to diseases later in life, particularly long-term lung function deficits and shortness of breath (Dauphine et al., 2011[4]). Nowadays, arsenic is a known carcinogen which has also been associated with various cancers including bladder, liver, lung, prostate, and skin cancer (Zhou and Xi, 2018[26]). Arsenic has also been associated with increased susceptibility to respiratory infections and cardiovascular disease. Interestingly, fetuses are extremely vulnerable to the effects of heavy metals, particularly arsenic (Lantz et al., 2009[10]; Vahter, 2009[19]; Ahsan et al., 2006[1]; Navas-Acien et al., 2005[14]).

A study in a Thai cohort has shown that arsenic exposure in utero increased the expression of genes involved in various responsive mechanisms such as apoptosis, stress responses, and inflammation (Fry et al., 2007[5]). Furthermore, DNA damage found in newborns indicated increased levels of urinary 8-nitroguanine, which is significantly correlated with an increased expression of inflammatory genes (COX2, EGR1, and SOCS3) in cord blood (Phookphan et al., 2017[15]).

A follow-up study in these prenatally arsenic exposed children showed an increase in oxidative and DNA damage, represented by increased levels of 8-Hydroxy-2'-deoxyguanosine and 8-nitroguanine, suggesting a risk of mutagenesis (Hinhumpatch et al., 2013[7]; Phookphan et al., 2017[15]). However, there are no firm conclusions as to whether the intake of low concentration arsenic contaminated drinking water adversely affects children's brains. An epidemiological study indicated that central nervous system (CNS) impairments such as cognitive or intellectual deficits were associated with arsenic exposure in children (Wasserman et al., 2007[23]; Nahar et al., 2014[13]; Tolins et al., 2014[18]). However, a study in West Bengal showed no association between long-term arsenic exposure in water and intellectual functions in children (von Ehrenstein et al., 2007[21]).

Over the past 10 years, arsenic contamination has been reported again in northern Thailand. The report on the health impact of the population within a 10 kilometer radius of a mine tailing in Phichit, Phitsanulok, and Phetchabun Provinces of Thailand by the Ministry of Public Health; Thailand, Forensic Science Institute Thammasat University and the Research Center to Promote Safety and the Prevent Injuries in Children Ramathibodi Hospital showed that more than 30 % of children (aged 8-13 years) had arsenic contamination higher than the normal range as indicated by the Agency for Toxic Substances and Disease Registry (ATSDR). After that, the mining was temporarily halted in 2016. 

Therefore, this study aimed to follow arsenic contamination after the mining industry had stopped operation for 3 years and to assess the health situation such as the nutrition status and intelligence quotient of children. Four approaches were used:

1) assess the nutrition status 

2) assess an intelligence quotient

3) assess the learning disability

4) assess an inorganic arsenic in urine

## Materials and Methods

### Study area

The areas within a 10 kilometer range of an inactive mining site (had stopped operation 3 years prior) in the northern part of Thailand were included in this study. All these areas had similar socioeconomic characteristics. 

### Study design

This was a cross-sectional research study and IRB approval was obtained from the Committee on Human Rights Related to Human Experimentation, Mahidol University, Salaya campus, Nakhon Pathom Province, Thailand (MU-CIRB 2019/067.2602).

### Subject

The subjects of this study comprised 199 school aged children (97 males and 102 females) from six elementary schools within a 10-kilometer radius of an inactive mining operation. The mean age of the subjects was 10.74 years (aged 10-12 years) (Supplementary Table 1). They were chosen by simple random sampling. All of them had been residents in these areas for at least 10 years.

### Nutritional status

Height and weight were measured for each student according to the WHO guidelines (WHO, 2006[24]). Weight was measured in kilograms and height in centimeters with one decimal point each. BMI percentiles were classified into four groups: underweight (<5^th^ BMI percentile), normal (5-84^th^ BMI percentile), overweight (85-94^th^ BMI percentile), and obese (≥95^th^ BMI percentile). 

### Intelligence quotient assessment

The subjects' intelligence quotient (IQ) was assessed by using the fourth edition of the test of nonverbal intelligence (TONI-4; Brown et al., 2010[3]) that requires no reading, writing, speaking, or listening in the examination. The test was administered to each child individually in a comfortable, well-lit, and noise-free room. 

### Learning disability assessment

Learning disabilities are caused by genetic and/or neurological factors that alter brain activity that affects learning processes and are not considered to be an intellectual disability. These processing problems can interfere with learning basic skills such as reading, writing, spelling, and math. The learning disability (LD) scores were assessed by using the KUS-SI Rating Scales: attention deficit hyperactive disorder (ADHD)/LD/Autism (Pervasive Developmental Disorders: PDDs) and it was found that the children had learning disabilities in at least one aspect (Kasetsart University Laboratory School: Center of Educational and Development/Department of Psychiatry, Faculty of Medicine Siriraj Hospital, Mahidol University). 

### Urinary samples collection

The first morning urine samples were collected from each subject and immediately kept on ice. Thererafter, all the urine samples were transferred to a liquid nitrogen tank and delivered to a laboratory.

### Inorganic arsenic assessment

The inorganic arsenic plus methylated metabolites-As (III), As (V), MMA, and DMA were analyzed by high performance liquid chromatography with inductively coupled plasma mass spectrometry (HPLC-ICP-MS). The values of inorganic arsenic were referenced by the Department of Medical Sciences, Ministry of Public Health, Thailand and ATSDR.

### Statistical analysis

For quantitative analysis, all data were expressed as means ± SD. The significance of differences in the data was evaluated by the t-tests. The relationship of data was calculated by the chi-square statistic. Significance was assumed at p<0.05.

## Results

### Nutritional status

The nutritional status of school-age children is demonstrated as follows: underweight 6.5 %, normal weight 63.4 %, overweight 5.0 %, and obesity 25.1 %, respectively. This study showed a statistically significant relationship between gender and nutritional status (Chi-square, p= 0.004; Table 1(Tab. 1)). Male children were twice as likely to be overweight and obese than female children. 

### IQ assessment

The IQs of children (TONI-4) were interpreted as follows: 

T score 69-70 = very poor

T score 71-79 = poor

T score 80-89 = below average

T score 90-109 = average

T score 110-119 = above average

T score 120-129 = superior

T score >129 = very superior.

The mean IQ score of 199 children was 92.1 ±10.0. The minimum and maximum IQ scores were 64 and 115, respectively. Male and female children had no statistically different IQ scores (p = 0.309, t-test). The children's IQ distribution is as follows: very poor 2.0 %, poor 9.0 %, below average 24.6 %, average 60.8 %, and above average 3.5 %, respectively. This study showed gender did not have any relationship with IQ (chi-square, p= 0.12; Table 2(Tab. 2); Supplementary Table 1). 

### LD assessment

This study found that 28 children had an LD. The mean of IQ score of children with an LD was 96.2 ± 6.3. The minimum and maximum IQ scores were 90 and 110, respectively. Of the 28, 27 children with LD (96.4 %) had a normal level of inorganic arsenic in their urine. However, one child (3.6 %) had a level of inorganic arsenic higher than the normal range (Table 3(Tab. 3)). 

### Analysis of inorganic arsenic in urine

**Figure 1:**
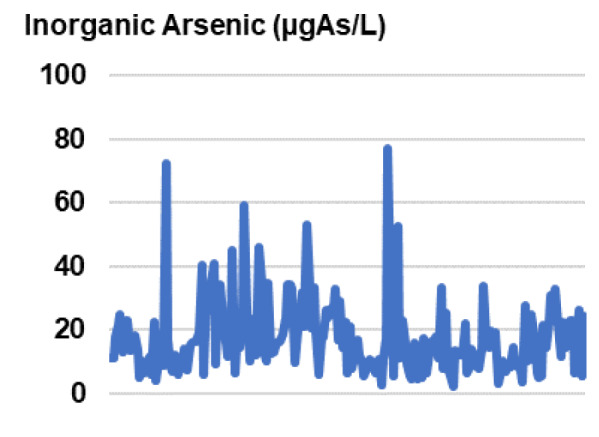
Level of inorganic arsenic in the urine of children (n=199). Assay was done by inductively coupled plasma mass spectrometry.

The mean of level of inorganic arsenic in urine was 17.69 ±11.82 µg As/L. The minimum and maximum were 2.50 and 77.0 µg As/L, respectively (Figure 1(Fig. 1)). The study found that 4.5 % of children had inorganic arsenic levels higher than the normal range as shown in Table 4(Tab. 4) and Supplementary Table 1.

**Figure 2:**
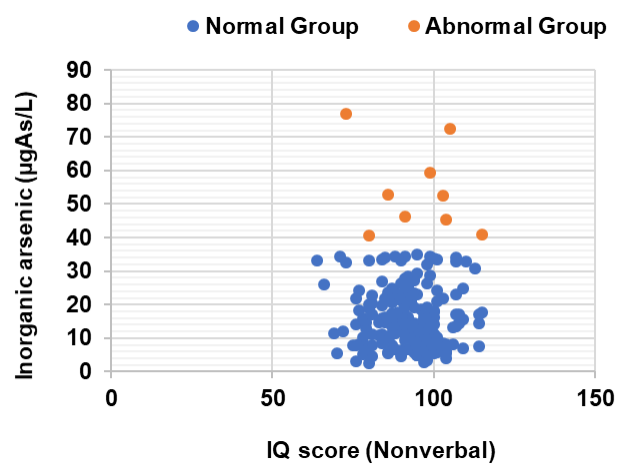
Scatter plot of inorganic arsenic level and IQ score of children (n=199). The study showed that the level of inorganic arsenic in urine did not have any relationship with IQ scores of children (Pearson correlation, p>0.05). The mean of IQ scores of children did not differ significantly between the two groups of inorganic arsenic: normal group and abnormal group (t-test, p>0.05). *Normal ranges of inorganic arsenic in urine are < 35 µg As/L as referenced by the Department of Medical Sciences, Ministry of Public Health, Thailand and ATSDR.

See also Figure 2(Fig. 2).

## Discussion

The most recent report provided by the ATSDR has suggested that toxic exposures to arsenic may result in memory loss and emotional instability in humans. Epidemiological studies from the past decade have provided a wealth of information supporting a strong correlation between arsenic exposure and neurological and cognitive dysfunction in children and adults. The clinical symptoms and pathology depend on concentration, timing, and duration of exposure; particularly, chronic arsenic exposure can induce various pathologies (Wang et al., 2007[22]; Roy et al., 2011[17]; Manju et al., 2017[11]). 

A current study of arsenic contamination in Thailand found that 4.5 % of school-age children had inorganic arsenic levels in their urine, higher than the normal range (ATSDR). This data showed that there is still inorganic arsenic contamination above the normal range in children living near an inactive mining site, even though the incidence is low.

The cause of contamination can be due to exposure, inhalation, and consumption. However, this study did not analyze any inorganic arsenic contamination in the environment, such as in the soil, in natural waters, or in food and drinking water. Therefore, the source of contamination cannot be identified in this study. 

Several studies have indicated that arsenic is contaminating the food chain, which can affect health and lead to chronic illnesses in children (Fry et al., 2007[5]; Rosado et al., 2007[16]; Dauphine et al., 2011[4]; Tolins et al., 2014[18]; Phookphan et al., 2017[15]; Manju et al., 2017[11]). We examined nutritional status and clinical symptoms of children. The results showed that 25.1 % of children were obese. Male children were twice as likely to be overweight and obese than female children. 

In addition, we did not find any clinical symptoms from arsenic contamination among all children in this study. However, more studies such as those on stress response, inflammation, DNA damage, and apoptosis are needed in the future. 

This study found that the mean of inorganic arsenic in urine was 17.69±11.81 µg As/L (below normal range) and level of inorganic arsenic in urine did not have any relationship with the IQ scores of children. Our study differed from several studies such as a 2007 study from Mexico, which found a significant relationship between urinary arsenic concentrations greater than 50 μg As/L and poor scores on intelligence tests of children aged 6-8 years (Rosado et al., 2007[16]). A 2007 study from China found that low exposure to arsenic (<10 μgAs/L urinary arsenic) resulted in no measurable effects on IQ. However, moderate and high exposure to arsenic (46 μg As/L and 73 μg As/L) resulted in decreases of 5 and 10 IQ points, respectively (Wang et al., 2007[22]).

A cohort study of children from Mexico (6-7 years old with 55 μg As/L urinary arsenic) showed poorer scores in arsenic exposed children on measures of language and vocabulary and a modest association with hyperactive behavior using the ADHD index (Roy et al., 2011[17]).

As mentioned above, a dose-dependent relationship existed between arsenic levels and poor performance scores on intelligence measures. Interestingly, several studies found that children who had a urinary arsenic level of more than 50 μg As/L will have a high risk of poorer intellectual performance (Wang et al., 2007[22]; Roy et al., 2011[17]; Manju et al., 2017[11]).

In addition, we found 1 LD child (3.6 %) who had inorganic arsenic levels above the normal range. However, more research is needed for LD groups because research on arsenic contamination of children with LD has rarely been done in Thailand.

Hence, the next phase of research should involve more studies being done, especially in Thailand. This would be useful in determining if the current cutoff (35 μg AS/L) will be sufficient to block arsenic-induced neurotoxicity, and if not, what can be done, either legislatively or through medical therapy to alleviate arsenic-induced neurotoxicity.

Therefore, the monitoring of heavy metal contamination in Thailand and any health effects in vulnerable children who live near the mining industry during normal operation or after it being temporarily suspended will prevent and mitigate health impacts that may occur in the future.

## Declaration

### Supplementary information

Supplementary information is available on EXCLI Journal website.

### Acknowledgments

This project is supported by the Health Systems Research Institute (HSR). We gratefully acknowledge all staff of the six schools which participated in the project. 

### Conflict of interest

The authors declare that they have no conflicts of interest.

## Supplementary Material

Suppplementary information

Supplementary data

## Figures and Tables

**Table 1:**
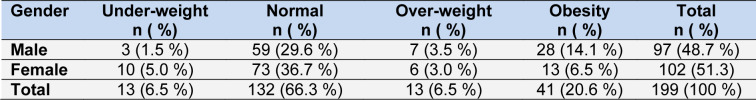
Number and percentage of nutritional status of children (n=199)

**Table 2:**
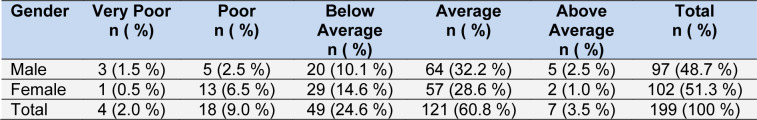
Number and percentage of child's intellectual ability (IQ) of children (n=199)

**Table 3:**
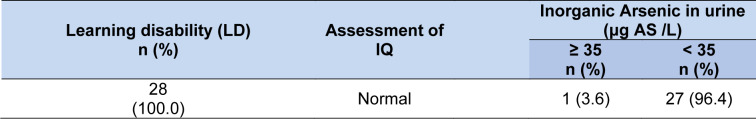
Number and percentage of inorganic arsenic in urine of 28 children that had a learning disability (LD)

**Table 4:**
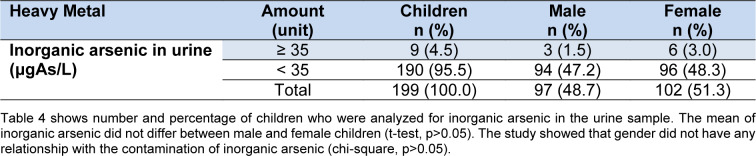
Number and percentage of children who were analyzed for inorganic arsenic levels in urine sample (n=199)
