# Supplementary material for: Inorganic arsenic contamination and the health of children living near an inactive mining site: northern Thailand
Source: EXCLI J. 2022 Jul 26;21:1007–14. doi: 10.17179/excli2022-4922 (PMC9441675; doi:10.17179/excli2022-4922)
Supplement: Suppplementary information [file EXCLI-21-1007-s-001.pdf]

EXCLI Journal 2022;21:1007-1014 – ISSN 1611-2156
Received: April 08, 2022, accepted: July 20, 2022, published: July 26, 2022

Supplementary information to:

Original article:

INORGANIC ARSENIC CONTAMINATION AND THE HEALTH OF CHILDREN LIVING NEAR AN INACTIVE MINING SITE: NORTHERN THAILAND

Sarun Kunwittaya[1], Nootchanart Ruksee[1], Thirata Khamnong[1], Athiwat Jiawiwatkul[1], Nonthasruang Kleebpung[1], Vasunun Chumchua[1], Adisak Plitponkarnpim[1,2], Chutikorn Nopparat[3], Kannika Permpoonputtana[1]*

[1] National Institute for Child and Family Development, Mahidol University, Nakhon Pathom 73170, Thailand
[2] Faculty of Medicine Ramathibodi Hospital, Mahidol University, Bangkok 10400, Thailand
[3] Innovative Learning Center, Srinakharinwirot University, Sukhumvit 23, Bangkok, 10110, Thailand

* **Corresponding author:** Kannika Permpoonputtana, National Institute for Child and Family Development, Mahidol University, Nakhon Pathom 73170, Thailand, Phone: (662) 441-0602; Fax: (662) 441-0167; E-mail: E-mail: kannika.per@mahidol.ac.th

https://dx.doi.org/10.17179/excli2022-4922

This is an Open Access article distributed under the terms of the Creative Commons Attribution License (http://creativecommons.org/licenses/by/4.0/).

Supplementary Table 1: Demographic characteristics of subjects

Variables	Total Sample (N=199)
**Age, years (Mean ± SD)**	10.74 ± 1.10
Sex, n (%)	
Male	97 (48.74 %)
Female	102 (51.26 %)
**Height, centimeters (Mean ± SD)**	141.46 ± 9.47
**Weight, kilograms (Mean ± SD)**	39.04 ± 13.96
**BMI, kg/m^2 (Mean ± SD)**	19.08 ± 4.93
**LD Assessment, n (%)**	
LD	28 (14.07 %)
Non-LD	171 (85.93 %)
**IQ score (Mean ± SD)**	96.25 ± 6.34
IQ level, n (%)	
Very poor	4 (2.01 %)
Poor	14 (7.04 %)
Below average	49 (24.62 %)
Average	125 (62.81 %)
Above average	7 (3.52 %)
**Urinary Inorganic Arsenic, µg As/L (Mean ± SD)**	17.69 ± 11.82

Data are presented as mean ± standard deviation or %; BMI, Body Mass Index; LD, Learning disability; IQ, Intelligence Quotient
